# Synthesis of bi-phase dispersible core-shell FeAu@ZnO magneto-opto-fluorescent nanoparticles

**DOI:** 10.1038/srep16384

**Published:** 2015-11-09

**Authors:** Xue-Mei Li, Hong-Ling Liu, Xiao Liu, Ning Fang, Xian-Hong Wang, Jun-Hua Wu

**Affiliations:** 1 Key Lab of Polyoxometalate Chemistry of Henan Province, Institute of Molecular and Crystal Engineering, School of Chemistry and Chemical Engineering, Henan University, Kaifeng 475001, China; 2 E-Techco Group, Shenzhen 518057, China; 3 Department of Materials Science and Engineering, South University of Science and Technology of China, Shenzhen 518055, China; 4 Pioneer Research Center for Biomedical Nanocrystals, Korea University, Seoul 136-713, South Korea

## Abstract

Bi-phase dispersible core-shell FeAu@ZnO magneto-opto-fluorescent nanoparticles were synthesized by a modified nanoemulsion process using poly(ethylene glycol)-block-poly(propylene glycol)-block-poly(ethylene glycol) (PEO-PPO-PEO) as the surfactant. The morphology and crystal structure of the nanoparticles were studied by TEM/HRTEM and XRD. The nanoparticles manifest soft ferromagnetic and/or near superparamagnetic behavior with a small coercivity of ~19 Oe at room temperature. The corresponding magnetic hysteresis curves were elucidated by the modified Langevin equation. The FTIR study confirms the PEO-PPO-PEO molecules on the surface of the nanoparticles. The UV-vis and PL results reveal the well-behaved absorption bands including surface plasmon resonance and multiple visible fingerprint photoluminescent emissions of the nanoparticles dispersed in both hydrophilic and hydrophobic solvents. Moreover, the processes of solvent dispersion-collection of the nanoparticles were demonstrated for application readiness of such core-shell nanostructures.

Multicomponent hybrid nanomaterials with tunable composition and morphology, have been used widely in the catalytic, optical, and magnetic research fields. By the coupling between the individual components, enhanced functionality or new properties can be provided in contrast to the more limited single-component counterparts, and their potential applications will be greatly expanded to cover more areas. Syntheses of such nanoparticles and investigating their properties are hence of great interest. On one hand, nanostructures made of iron and gold in the past several years have been subjects of intensive research due to their hybrid nature and potential applications in a variety of fields, such as nanodevices, biomedicine and physical sciences^1^,^2^,^3^,^4^,^5^. In addition to the magnetic properties of Fe, FeAu nanostructures exhibit surface plasmon resonance (SPR) properties as a result of incorporation of Au^1^,^6^,^7^. On the other hand, ZnO is a wide bandgap (3.37 eV) semiconductor material with a large exciton binding energy (60 meV) at room temperature, manifesting many attractive properties, including unique optical, acoustic, and piezoelectric effect^8^,^9^,^10^,^11^,^12^.

Therefore, nano-engineering the FeAu and ZnO into a single entity would not only exercise the unique properties of FeAu and the ZnO semiconductor, but also generate novel phenomena based on the interaction between FeAu and ZnO at their interfaces. Such nano-assembly can lead to innovative materials which exhibit more broad application prospects owing to their novel optical, magnetic, electrical, and chemical properties. In this work, bi-phase dispersible FeAu@ZnO magneto-opto-fluorescent core-shell nanoparticles were synthesized by a modified nanoemulsion process using poly(ethylene glycol)-block-poly(propylene glycol)-block-poly(ethylene glycol) (PEO-PPO-PEO) as the surfactant.

The PEO-PPO-PEO triblock copolymer and families encompass numerous distinct advantages such as biocompatibility, non-charging trait, non-toxicity, and aqueous solubility, and are frequently used in diverse fields^13^,^14^,^15^,^16^,^17^. In nanoemulsion processes, the PEO-PPO-PEO molecules participate in the reactions as the surfactant, playing a role in stabilizing the nanoparticles formed, and even acting as a reducing agent. We have investigated a number of multi-functional nanoparticles using PEO-PPO-PEO molecules as the surfactant, such as long-term stable, monosized, highly crystalline FeAu, ZnO-Au and AgZnO nanoparticles^6^,^18^,^19^. The PEO-PPO-PEO-laced FeAu@ZnO nanoparticles as prepared in this work show high crystallinity, monodispersity, bi-phase dispersibility, excellent optical performance both in organic and aqueous medium, and outstanding magnetic response. The magnetic hysteresis curves of the nanoparticles were elucidated by the modified Langevin equation, demonstrating the well-defined soft ferromagnetic and/or near superparamagnetic behavior of the core-shell nanoparticles at room temperature. Such bi-phase dispersible core-shell FeAu@ZnO magneto-opto-fluorescent nanoparticles could be of interest for fundamental studies and potential applications.

## Results and Discussions

**Figure 1:**
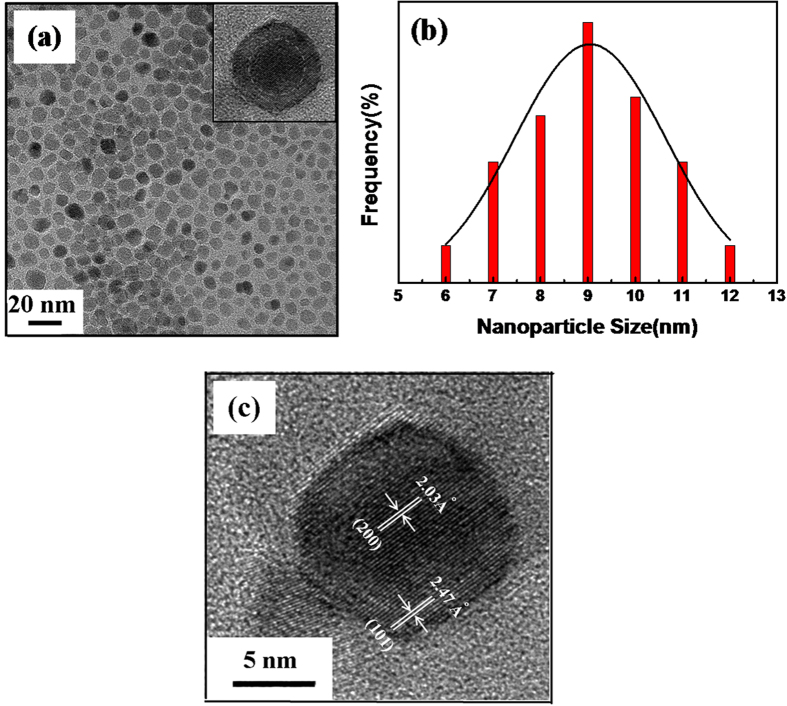
TEM analyses of the PEO-PPO-PEO-laced FeAu@ZnO core-shell nanoparticles. (**a**) Bright-field image, (**b**) particle size histogram with Gaussian fit, (**c**) HRTEM of an individual FeAu@ZnO nanoparticle.

The morphology, nanostructure, particle size and size distribution of the PEO-PPO-PEO-laced FeAu@ZnO nanoparticles were recorded by TEM, HRTEM and XRD. As given in Fig. 1a, the obtained FeAu@ZnO nanoparticles are highly crystalline, virtually uniform, and spherical in shape, with the inset showing an example of the core-shell configuration. The size distribution of the nanoparticles is presented in Fig. 1b, in which the histogram shows a tight size distribution and an average size of ~9.0 nm in diameter. The distribution can be reasonably described by a Gaussian function. Figure 1c represents the HRTEM image of a single FeAu@ZnO core-shell nanoparticle. As labeled, the spacing of 2.03 Å is corresponding to the (200) reflection of the FeAu face-centered cubic (fcc) phase^6^, whereas the spacing of 2.47 Å is originated from the (101) reflection of the ZnO hexagonal wurtzite structure. The observation clearly illustrates the formation of core-shell nanoparticles. The core-shell FeAu@ZnO nanoparticles are thus highly crystalline, as consolidated in the X-ray analysis below.

**Figure 2:**
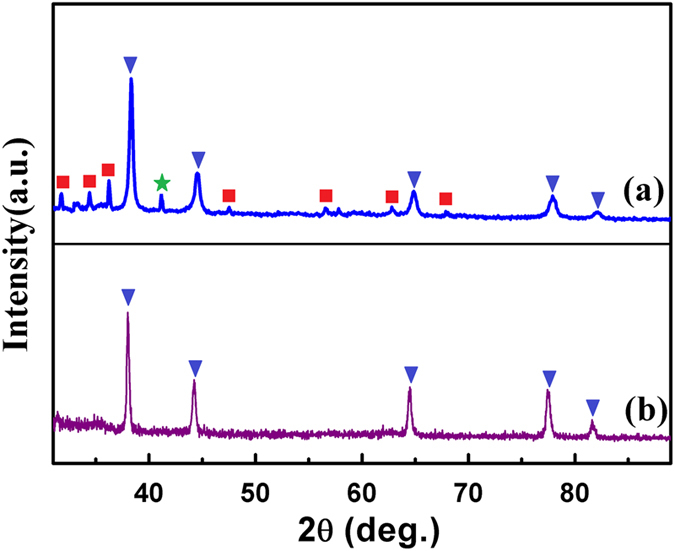
XRD patterns of the nanoparticles. (**a**) FeAu@ZnO, (**b**) FeAu nanoparticles.

Figure 2 compares the XRD pattern of the PEO-PPO-PEO-laced FeAu@ZnO core-shell nanoparticles with that of the FeAu nanoparticles prepared by analogous synthesis methods^6^. As shown in Fig. 2b, the diffraction peaks positioning at 38.19°, 44.31°, 64.70°, 77.64° and 81.61° are indexed to the (111), (200), (220), (311), and (222) planes of the FeAu nanoparticles^6^. The XRD pattern for the FeAu@ZnO core-shell nanoparticles is given in Fig. 2a. In contrast to the FeAu nanoparticles, the FeAu@ZnO core-shell nanoparticles simultaneously preserve highly crystalline features of both FeAu and ZnO component materials. The distinct diffraction peaks as labeled by inverted triangles are indexed to the (111), (200), (220), (311) and (222) planes of the FeAu, which are the same as that of the FeAu nanoparticles (Fig. 2b), while the other diffraction peaks as labeled by squares correspond to the (100), (002), (101), (102), (110), (103) and (112) planes of the ZnO hexagonal wurtzite (JCPDS no. 36–1451). In addition, the diffraction peak at 40.94° as labeled by star arises from the AuZn_3_ (033) plane. The observation that the peaks from ZnO are weaker than those from FeAu is most likely attributed to the heavy atomic effect of Au^6^. The XRD outcome is suggestive of the formation of the core-shell nanostructure. Moreover, based on the full width at half maximum (FWHM) of the corresponding most intense diffraction peaks by using the Debye-Scherrer equation, the average particle size of the FeAu@ZnO nanoparticles is calculated to be ~9.4 nm, comparable to that from the statistical size counting of the TEM analysis above, supposing that the broadening of the peaks in the XRD pattern is predominantly due to the finite size effect of the nanoparticles^20^.

**Figure 3:**
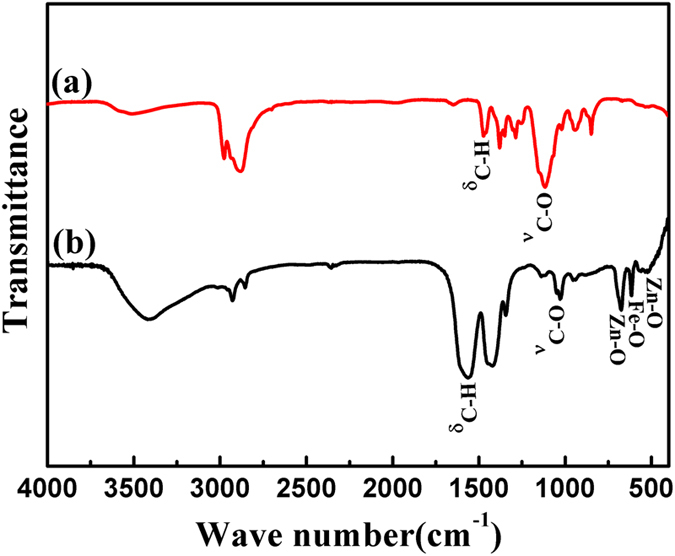
FTIR spectra of (**a**) the pure PEO-PPO-PEO polymer, and (**b**) the PEO-PPO-PEO-laced FeAu@ZnO nanoparticles.

To establish the presence of the PEO-PPO-PEO macromolecules on the surface of the FeAu@ZnO core-shell nanoparticles in this work, FTIR measurements were conducted on the purified nanoparticles and the pure polymer itself. In Fig. 3a, the pure PEO-PPO-PEO polymer molecules display one strong characteristic band at the position of approximately 1110.82 cm^−1^ belonging to the C–O–C stretching vibration of the ether bonding which usually ranges between 1250 cm^−1^ and 1000 cm^−1^ and one sharp characteristic band due to the C–H bending vibration at the position of 1467.36 cm^−1^. As given in Fig. 3b, these characteristic vibrations and bending features reappear in the FTIR spectrum of the PEO-PPO-PEO-laced FeAu@ZnO core-shell nanoparticles, but redshifted to the corresponding positions of 1032.59 cm^−1^ for the C–O–C stretching vibration and blue-shifted to 1562.89 cm^−1^ for the C–H bending vibration, respectively. Additionally, the band shapes and absorption intensities differ noticeably from the pure PEO-PPO-PEO molecules to the core-shell nanoparticles. In mechanism, the shifting and changes in the C–O–C stretching and C–H bending modes may be due to the coordination of the oxygen atoms in the PEO-PPO-PEO main chains to the Zn atoms in the core-shell nanostructure which could be attributed to changes in the elastic constants of the bonds of the macromolecules sitting on the nanoparticle surface of high curvature because of the small nanoparticle size and interactions between the macromolecules and the nanoparticle surface^16^,^18^. Furthermore, the symmetric deformation band of methyl groups in the spectra is observed at 1380.11 cm^−1^ for the pure PEO-PPO-PEO molecules, but blue-shifted to 1423.41 cm^−1^ for the PEO-PPO-PEO-laced nanoparticles^21^. Meanwhile, the double C–H stretching vibrations in the spectra are positioned at 2875.47 and 2980.73 cm^−1^ for the pure PEO-PPO-PEO molecules, but redshifted to 2858.17 and 2927.31 cm^−1^ for the PEO-PPO-PEO-laced nanoparticles, respectively^16^. Still, the peaks at 527.87 cm^−1^ and 676.24 cm^−1^ observed in the spectrum of the PEO-PPO-PEO-laced FeAu@ZnO core-shell nanoparticles may occur thanks to the Zn–O vibration^22^,^23^, and the peak at 615.32 cm^−1^ pertain to Fe–O vibration^24^. Consequently, the observation provides strong evidence for the covering of the PEO-PPO-PEO macromolecules on the surface of the FeAu@ZnO nanoparticles, as the redundant PEO-PPO-PEO molecules were removed by the washing procedure. Owing to the PEO-PPO-PEO lacing, therefore, the FeAu@ZnO nanoparticles turn out to be both hydrophobic and hydrophilic, which enables an easy transfer of the nanoparticles between non-polar and polar solvents without additional surface decoration. As often nanoparticles as synthesized are of necessity to be transferred to and processed in an aqueous medium in applications of nanobiotechnology and nanomedicine, the natural coating of nanoparticle surfaces by an ultrathin film of biocompatible PEO-PPO-PEO molecules is highly desirable for future biomedical applications, which is one of the purposes as described in this work.

**Figure 4:**
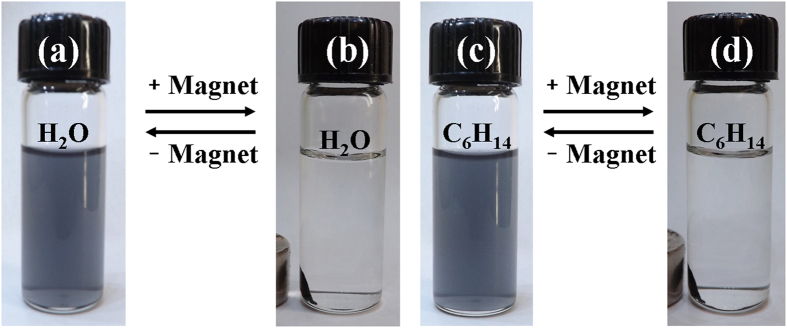
Photoimages of solvent dispersion-collection process of the PEO-PPO-PEO-laced FeAu@ZnO nanoparticles.

Figure 4 visually demonstrates the separation and redispersion process of the nanoparticles in water and hexane. Under the influence of an external magnetic field, the FeAu@ZnO nanoparticles in water change from a purplish grey, homogeneous dispersion (Fig. 4a) to a clear, transparent solution, with the nanoparticles collected by a piece of magnet (Fig. 4b). The collected nanoparticles can be easily and reversibly dispersed by agitation after removal of the magnetic field and the above process can be repeated. A similar process happens to the nanoparticles in hexane, as shown in Fig. 4c,d. The color of the solution in Fig. 4a,c is complementary to the corresponding UV-vis absorption to be discussed below. The finding that all PEO-PPO-PEO-laced FeAu@ZnO nanoparticles as prepared could be collected by a magnet, leaving no free Au and ZnO particles visible and thus no co-sedimentation of Au, ZnO and Fe nanoparticles excludes the possibility of separate Au, ZnO and Fe nanoparticles in the FeAu@ZnO preparation and supports the nanostructuring of the FeAu@ZnO nanoparticles. In other words, we performed magnetic separation and found that all nanoparticles were collected by the magnet because the PEO-PPO-PEO-laced FeAu@ZnO core-shell nanoparticles are magnetic and there is no more nanoparticles left-over, so nonmagnetic ZnO nanoparticles are not present in the samples.

**Figure 5:**
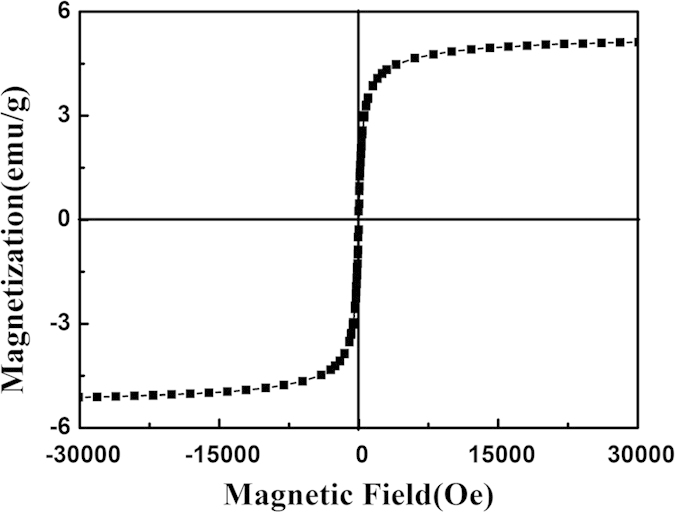
Hysteresis curve of the PEO-PPO-PEO-laced FeAu@ZnO nanoparticles recorded at room temperature.

The magnetic properties of the PEO-PPO-PEO-laced FeAu@ZnO core-shell nanoparticles were studied by VSM at room temperature. As the PEO-PPO-PEO macromolecules are nonmagnetic in nature, thus the magnetism of the nanosystem is depended on the FeAu@ZnO ingredient which is in turn protected by PEO-PPO-PEO from oxidation. As presented in Fig. 5, the PEO-PPO-PEO-laced FeAu@ZnO core-shell nanoparticles show soft ferromagnetic or near superparamagnetic properties with a small coercivity of ~19 Oe and a magnetization of 5.2 emu/g at ~3 T. Mechanistically, the magnetic property of the FeAu@ZnO nanoparticles is subject to the spin-glass behavior in Fe-Au-ZnO systems, in addition to other factors such as nanostructuring, heterogeneous composition distribution, increased surface area, finite-size effects, surface magnetism, induced magnetism in nanostructured Au, spin–spin and spin–orbital interactions. Interesting information may be gained by the description of magnetization processes, which has been extended to the case of the PEO-PPO-PEO-laced FeAu multifunctional nanoparticles using a modified Langevin function in consideration of setting-in of the coercivity^6^,^25^,^26^,


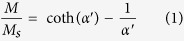


where


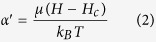


**Figure 6:**
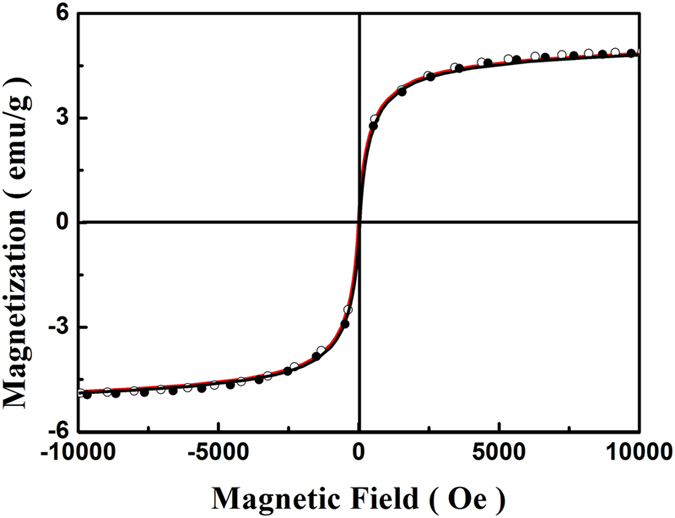
Analysis of the hysteresis curves of the PEO-PPO-PEO-laced FeAu@ZnO nanoparticles. Experimental data in curves and the fitting results in dots.

In the equations, *M/M*_S_ is the magnetization (*M*) normalized to the saturation magnetization (*M*_S_), *H* the applied magnetic field, *H*_c_ the coercivity, *k*_B_ the Boltzmann constant and *T* the temperature. The magnetic moment *μ* of the individual particles can be obtained from fitting of the modified Langevin function to the experimental data. As presented in Fig. 6, an initial analysis shows that the curves of the PEO-PPO-PEO-laced FeAu@ZnO core-shell nanoparticles can be reasonably elucidated by equation (1). The almost perfect single-phase fitting suggests that the PEO-PPO-PEO-laced FeAu@ZnO multifunctional nanoparticle system is likely composed of single magnetic phase^6^.

**Figure 7:**
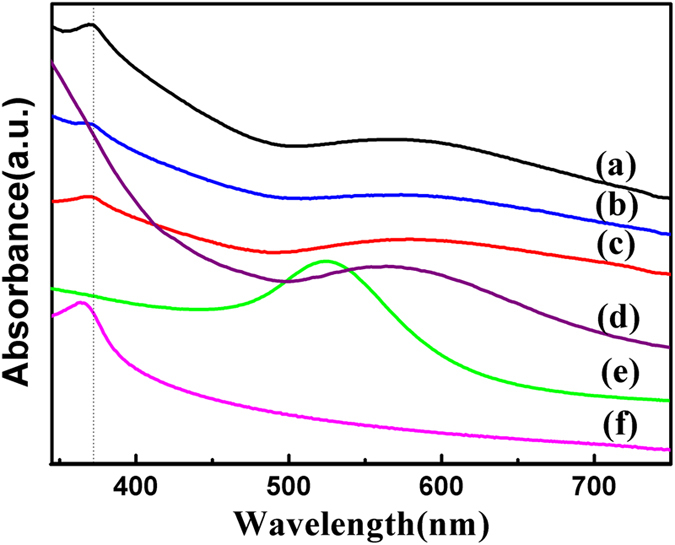
UV-visible absorbance spectra of the PEO-PPO-PEO-laced FeAu@ZnO dispersed in different solvents. Ethanol (**a**), hexane (**b**), and water (**c**), in comparison to FeAu (**d**), Au (**e**) and ZnO (**f**) nanoparticles (all in hexane).

The optical properties of the PEO-PPO-PEO-laced FeAu@ZnO core-shell nanoparticles were studied by UV-visible absorption spectroscopy and PL spectrometry, respectively. Figure 7 compares the UV-vis spectra of the FeAu@ZnO nanoparticles dispersed in ethanol (a), hexane (b), and water (c), together with those of FeAu (d), Au (e) and ZnO (f) nanoparticles in similar sizes dispersed in hexane. Two kinds of absorption bands are observed for the FeAu@ZnO core-shell nanoparticles, one from ZnO and the other from the surface plasmon resonance (SPR) of Au nanostructuring. The strong absorption bands observed around 373 nm, 372 nm, and 373 nm for the spectra of the FeAu@ZnO nanoparticles dispersed in ethanol (Fig. 7a), hexane (Fig. 7b) and water (Fig. 7c), respectively, are assigned to the most characteristic band of ZnO wide bandgap semiconductor material^27^, indicating redshifts with respect to the absorption peak of the ZnO nanoparticles in hexane at the position of approximately 364 nm, as shown in Fig. 7f. The redshifting of the core-shell nanoparticles may be in part attributed the formation of a ZnO nucleation layer on the surface of FeAu nanoparticle^28^. The peak position of the surface plasmon bands in the solution of the FeAu@ZnO nanoparticles varies from 575 nm in ethanol, 575 nm in hexane, to 577 nm in water, showing redshifts with respect to the absorption peak of the Au nanoparticles in hexane at the position of approximately 525 nm as shown in Fig. 7e, as well as the absorption peak of the FeAu nanoparticles in hexane at the position of approximately 566 nm as shown in Fig. 7d. The distinct redshifting and broadening of the SPR of the FeAu@ZnO core-shell nanoparticles compared with Au and FeAu nanoparticles substantiate the optical property of the FeAu@ZnO core-shell nanostructure. The well-defined absorption between 500 and 750 nm features the optical property of surface plasmon resonance as a consequence of Au nanostructuring^19^,^25^. After the formation of the FeAu core and then the covering ZnO shell, the absorption peak of Au observed at 525 nm is redshifted to 566 nm and then to 575–577 nm. Technically, the peak position and band shape of the plasmon resonance may be dependent on factors of composition, dimension, shape, dielectric medium and nanostructuring of the nanoparticle system^29^. The shifting of the SPR of the FeAu nanoparticles comparing with Au nanoparticles may be interpreted in terms of the electron cloud oscillation of Au atoms perturbed by the Fe atoms^1^. In contrast, the noticeable redshifting of the SPR of the FeAu@ZnO core-shell nanoparticles against the FeAu nanoparticles could be ascribed to the higher refractive index of the ZnO shell material in comparison to that in ethanol, hexane and water, indicating the formation of the ZnO shell on the FeAu core^30^,^31^,^32^. We remark that the broad plasmon resonance of the PEO-PPO-PEO-laced FeAu@ZnO core-shell nanoparticles of a reasonable intensity could have an advantage over the narrow optical responses of Au and FeAu nanoparticles, as the tight plasmon resonance of the latter could suffer from background interference.

**Figure 8:**
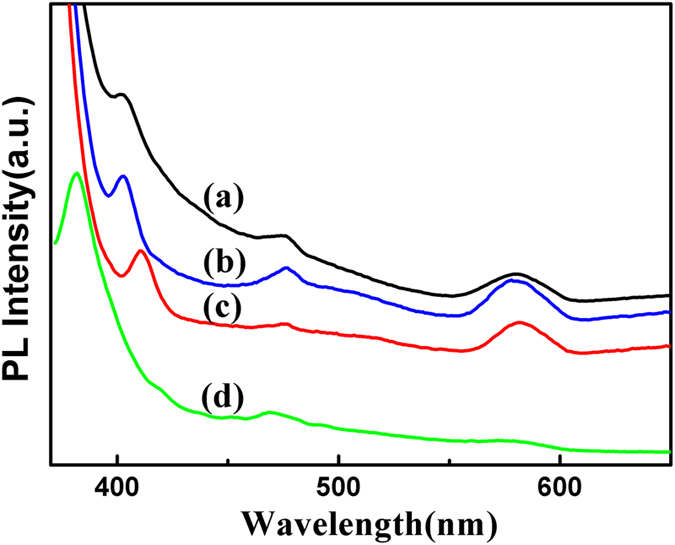
Photoluminescence emission spectra of the PEO-PPO-PEO-laced FeAu@ZnO nanoparticles dispersed in different solvents. Ethanol (**a**), hexane (**b**), and water (**c**), in comparison to ZnO nanoparticles dispersed in hexane (**d**).

As shown in Fig. 8, the PL emission spectra of the PEO-PPO-PEO-laced FeAu@ZnO core-shell nanoparticles dispersed in ethanol, hexane and water, respectively, in comparison to that of ZnO nanoparticles dispersed in hexane, were examined under the excitation wavelength of 360 nm. The UV emission bands around 403, 402 and 410 nm observed for the PEO-PPO-PEO-laced FeAu@ZnO core-shell nanoparticles dispersed in ethanol (Fig. 8a), hexane (Fig. 8b) and water (Fig. 8c), respectively, attributed to the near-band edge (NBE) emission from the recombination of free excitons^27^, showing redshifting with respect to that of the ZnO nanoparticles at the position of approximately 382 nm. The weak blue emission bands at around 476, 476, and 475 nm in ethanol, hexane and water, respectively, most likely originate from intrinsic defects such as oxygen and zinc interstitials, indicating a slight redshift compared to that of the ZnO nanoparticles at the position of approximately 468 nm^27^,^33^. The relatively strong green-yellow emission bands from the core-shell nanoparticles at around 580 nm in hexane, 579 nm in ethanol and 581 nm in water, against an almost indiscernible emission peaking at approximately 575 nm of the ZnO nanoparticles, are normally originated from the deep-level emissions, which were closely related with structural defects such as oxygen vacancies, zinc interstitials or surface and interface imperfection^34^,^35^. Moreover, the core-shell nanostructuring of the ZnO shell sitting on the FeAu core as well as possible doping of Fe and Au into the ZnO matrix could introduce stress, resulting in lattice deformation which may cause ZnO band structure deformation and thus the observed shifts of the UV, blue and green-yellow emission bands to lower energies, yet more delicate investigation remains useful for comprehensive understanding of the optical mechanism^36^,^37^,^38^. Furthermore, the redshift observed in UV emission bands of the FeAu@ZnO nanoparticles dispersed in water compared to those in ethanol and hexane could be due to the higher defects concentration in the aqueous mediated nanocomposite.

In short, the FeAu@ZnO core-shell nanoparticles show strong emissions at 402–410 nm, relatively weak emissions at around 475–476 nm and strong emissions at 579–581 nm. The shapes of the emission peaks are similar to the findings in the previous investigation on ZnO, Cu-ZnO, AgZnO and ZnO-Au nanoparticles, in both pure and hybrid forms^18^,^19^,^39^,^40^.

In order to determine the efficiency of emission quantitatively, the fluorescence quantum yield (QY) of the FeAu@ZnO nanoparticles in comparison to that of the ZnO nanoparticles were measured using a solution of Rhodamine 6G in ethanol (QY = 95%) as a reference material^41^,^42^. The result shows that the QY value of the violet-ultraviolet emission (375–420 nm) was 20.6% and 23.1% for the FeAu@ZnO and ZnO nanoparticles, respectively. As fluorescence QY represents the fraction of the excited molecules to lose energy through fluorescence, the FeAu@ZnO core-shell nanoparticles as observed reveal a relatively reasonable QY of the violet-ultraviolet emission together with the green-yellow emission which is benign to application^43^. However, the fluorescence is a delicate process that the photonic emission competes with other energy dissipation paths and the surface state of ZnO nanoparticles may play a leading role in determining their visible fluorescence^44^,^45^,^46^,^47^. The relatively high quantum yield of the violet-ultraviolet emission for the FeAu@ZnO and ZnO nanoparticles may be partially due to the laced PEO-PPO-PEO polymers which are used to coordinate with Zn atoms on the surface of ZnO nanoparticles to hinder the formation of bulk ZnO. Nonetheless, the contributions of the FeAu core to the PL emissions may be comprehended in several aspects. Mechanistically, the rich free electrons in the FeAu core nanocrystals stimulate the electronic density waves that have their own wavelength depending on the size and shape. Appraising Fig. 7a–c, the peak positions of the SPR resonance and the PL emissions are comparable. As a consequence, two opposing mechanisms arise. In one hand, the electrons in the defect level of ZnO can be excited to the conduction band by the energy transfer via the SPR mode of the FeAu core nanocrystals activated by the incident electromagnetic waves so that the exciton density rises and therefore, the probability of the relevant emissions is enhanced. On the other hand, the emitted photons may be reabsorbed by the FeAu core nanocrystals through exciting surface plasmon waves. Such energy dispersion reduces the corresponding PL emission. Yet, the lower QY value of the violet-ultraviolet emission from the FeAu@ZnO nanoparticles compared to that of the ZnO nanoparticles may be caused by the quenching interaction between the metallic FeAu core and the semiconducting ZnO shell in which electrons may transfer from FeAu to ZnO, as well as the increased zinc interface defects and the reduced probability of surface-trapped holes as a result of the nanostructuring^35^,^37^,^38^. We state that many factors can play a significant role in the quenching and enhancement mechanisms of photoluminescence, and their effects are still in debate. A proper exposition of the mechanisms is of importance and challenging, particularly true for a complex system such as the present case which requires more investigation.

We point out that the fact that ZnO possesses environmentally friendly features for photocatalytic applications. But, due to the high rate of electron-hole recombination and visible blindness, however, its applications are somewhat restricted. Nano-engineering both FeAu core and ZnO shell into a single entity could tune down the electron-hole recombination correspondingly and then substantially increases the photocatalytic activity of the material, in addition to easy separation and recovery from the reaction medium as a consequence of the magnetic composition.

## Conclusions

In summary, we successfully synthesized the PEO-PPO-PEO-laced FeAu@ZnO magneto-opto-fluorescent core-shell nanoparticles via nanoemulsion process adopting the biocompatible and non-toxicity triblock copolymer PEO-PPO-PEO as the surfactant. The morphology and crystal structural analyses illustrate that the FeAu@ZnO nanoparticles reveal high crystallinity, virtually uniform, spherical in shape and narrow particle size distribution with an average size of ~9 nm. The PEO-PPO-PEO-laced FeAu@ZnO core-shell nanoparticles show soft ferromagnetic and/or near superparamagnetic properties with a small coercivity of ~19 Oe, and the corresponding magnetic hysteresis curves were elucidated by modified Langevin equations. The FTIR assessment shows that PEO-PPO-PEO molecules are present on the surface of the nanoparticles. The UV-vis and fluorescence measurements show the well defined optical absorption and emission properties of the nanoparticles. The bi-phase dispersible FeAu@ZnO magneto-opto-fluorescent nanoparticles may offer exciting opportunities in optical sensing, medical diagnostics, or photocatalysis.

## Methods

### Synthesis of the PEO-PPO-PEO-laced FeAu@ZnO core-shell nanoparticles

The FeAu@ZnO nanoparticles were prepared by the controlled sequential synthesis of the ZnO capping onto the surface of the FeAu seeds. In a typical experiment, Fe(acac)_3_ (0.5 mmol) in 10 ml octyl ether was mixed with Au(OOCCH_3_)_3_ (0.5 mmol), the reduction agent 1,2-hexadecanediol (5 mmol) and the surfactant PEO-PPO-PEO (0.5 g) in a 100 ml flask. Under vigorous stirring, the reaction mixture was first heated to 80 °C and homogenized at that temperature for 2 h, then rapidly raised to 280 °C and refluxed at that temperature for 1 h to complete the formation of the FeAu nanoparticles. After the reaction mixture was cooled to room temperature, 3 mmol of Zn(acac)_2_, 3 mmol of 1,2-hexadecanediol and 2 ml of dioctyl ether were added to the flask. Under vigorous stirring, the reaction mixture was first heated to 80 °C and homogenized at that temperature for 1 h, then rapidly heated to 280 °C and refluxing for 1 h at 280 °C to allow the ZnO shell build-up on the surface of the FeAu nanoparticles. To ensure the compactness of the ZnO nanolayer, the shell building step was repeated two times analogously except for the various amount of precursor Zn(acac)_2_ 5 mmol and 8 mmol respectively. After cooling down to room temperature, the precipitated product was washed with the mixed ethanol/hexane (1:2) several times, and re-dispersed in hexane for future use. For comparison, ZnO and Au nanoparticles were prepared similarly using only Zn(acac)_2_ and Au(OOCCH_3_)_3_ as the precursor respectively.

### Characterization of the PEO-PPO-PEO-laced FeAu@ZnO core-shell nanoparticles

The particle size and morphology of the PEO-PPO-PEO-laced FeAu@ZnO core-shell nanoparticles were studied by transmission electron microscopy (TEM, JEM-100CX) including the mode of high resolution (HRTEM), while the crystal structure of the nanoparticles was acquired by X-ray powder diffraction (XRD, X’Pert Pro). The Fourier transform infrared spectroscopy (FTIR) studies of FeAu@ZnO nanoparticles and pure PEO-PPO-PEO polymer were performed using an Avatar 360 FTIR spectrometer (Nicolet Company, USA). The optical properties of the samples were analyzed by an UV-visible spectrometer (UV-vis near IR spectrophotometer, Hitachi U4100) and a photoluminescence (PL) spectrophotometer (Hitachi F7000, Japan). The relative quantum yields (QYs) were measured using a solution of Rhodamine 6G in ethanol (QY = 95%) as a reference material^41^,^42^. In addition, vibrating sample magnetometry (VSM, Lakeshore 7300) was applied to perform the magnetic measurements on the dried samples to evaluate the magnetic properties of the core-shell nanoparticles at room temperature. The dispersion-collection processes of the FeAu@ZnO nanoparticles both in water and hexane were visually demonstrated for application readiness.

### Magnetic analysis of the hysteresis curves

A modified Langevin equation was employed to analyze the hysteresis curves of the PEO-PPO-PEO-laced FeAu@ZnO core-shell nanoparticles.

## Additional Information

**How to cite this article**: Li, X.-M. *et al.* Synthesis of bi-phase dispersible core-shell FeAu@ZnO magneto-opto-fluorescent nanoparticles. *Sci. Rep.* **5**, 16384; doi: 10.1038/srep16384 (2015).
